# Laboratory efficacy of selected synthetic insecticides against second instar invasive fall armyworm, *Spodoptera frugiperda* (Lepidoptera: Noctuidae) larvae

**DOI:** 10.1371/journal.pone.0265265

**Published:** 2022-05-16

**Authors:** Atif Idrees, Ziyad Abdul Qadir, Ayesha Afzal, Qiu Ranran, Jun Li

**Affiliations:** 1 Guangdong Key Laboratory of Animal Conservation and Resource Utilization, Guangdong Public Laboratory of Wild Animal Conservation and Utilization, Institute of Zoology, Guangdong Academy of Sciences, Guangzhou, China; 2 Honeybee Research Institute, National Agricultural Research Centre, Islamabad, Pakistan; 3 Department of Entomology and Wildlife Ecology, University of Delaware, Newark, Delaware, United States of America; 4 Institute of Molecular Biology and Biotechnology, The University of Lahore, Lahore, Pakistan; Anhui Agricultural University, CHINA

## Abstract

Maize is the most essential crop of China and its productivity has been recently endangered by the fall armyworm (FAW), *Spodoptera frugiperda*. Chemical pesticides are one of the most important strategies for managing FAW on a short-term basis. The seven synthetic insecticides including novel and conventional belong to four chemical group, spinetoram and spinosad (spinosyns), lambda-cyhalothrin, cypermethrin and bifenthrin (pyrethroids), abamectin (avermectins), broflinilide (diamides), were assessed for their efficiency in causing mortality to second instar *S*. *frugiperda* larvae at 24, 48 and 72 h post-treatment at five different serial concentrations (10 to 0.625 mg liter^-1^). The second instar *S*. *frugiperda* larvae were susceptible to the tested synthetic insecticides, however, the toxicity index of synthetic insecticides was estimated based on lethal concentration 50 (LC_50_), while, LC_50_ was calculated from the data of larval mortality. The broflanilide and abamectin proved to be the most toxic having the highest toxicity index of 100 and 78.29%, respectively, followed by cypermethrin and bifenthrin were showed toxicity index of 75.47 and 66.89%, respectively. The LC_50_ values were 0.606 and 0.774 mg liter^-1^ for broflanilide and abamectin, respectively, followed by cypermethrin and bifenthrin were showed LC_50_ values of 0.803 and 0.906 mg liter^-1^ at 72 h post-treatment. Rest of the other synthetic insecticides were showed moderate toxicity index of 42.11 to 62.09%, based on LC_50_ values were 1.439 to 0.976 mg liter^-1^ at 72 h post-treatment. The efficiency of synthetic insecticides was increased by increasing concentration level and exposure time. The screened synthetic insecticides among seven insecticides perhaps, provide basis for the development of novel insecticides for controlling *S*. *frugiperda* population after further research to evaluate and validate the laboratory results in the field.

## Introduction

Maize is one of the staple crops of China [1], after the United States, China is the world’s second-largest maize producer and consumer [2]. Northeast China is China’s leading grain-producing region, producing one-fifth of the country’s grain [3]. *Spodoptera frugiperda* (J.E. Smith) (Lepidoptera: Noctuidae), the fall armyworm, is a prolific, polyphagous insect pest of 350 host plant species, primarily maize (*Zea may*s L.) [4, 5], and native to the Americas. *S*. *frugiperda* has been spread over the world and was detected for the first time in Africa (2016), Nepal and Indonesia in 2019 [6, 7], while a rice strain of FAW was recently identified in Swaziland [8]. *S*. *frugiperda* has emerged as one of the most severe threats to agricultural crop production, negatively affecting cereal and vegetable crops worldwide [9]. In many countries, food security and livelihood are threatened by the recent invasion of this new pest [10], and are widespread in more than 100 countries [11]. *S*. *frugiperda* larvae can be found on young leaves, leaf whorls, tassels, or cobs, depending on their stage of development. Damage occurs in skeletonized leaves and severely windowed whorls in the first instar larvae, who scrape leaves and display pin-hole symptoms and window-pane eating symptoms, but damage results in the later vegetative stages in skeletonized leaves and window-pane feeding symptoms. If the environmental conditions for pest establishment are favorable, this insect might cause a 100% crop loss in maize if it is not controlled on time [12, 13].

Maize remains a crucial part of the food security equation in maize producing countries for both human and animal consumption. *S*. *frugiperda*, on the other hand, poses a major threat to maize at all phases of its development [13], caused economic harm to maize crops in more than 44 African nations by 2019, resulting in yearly yield losses of US$2.5–6.2 billion [14]. Currently, *S*. *frugiperda* is classified as an A1 quarantine pest, and agricultural goods are subject to strict cross-border controls [15]. In India, fodder maize was found to have a damage rate of 16 to 52% [16]. Furthermore, most maize growers in Ethiopia and Kenya (93 and 97%, respectively) reported *S*. *frugiperda* damage in their fields, with production losses of up to 100%. As a result, most maize producers prefer to tackle this invasive insect with synthetic pesticides [17], most of the farmers (60%) believed that pesticides were ineffective for suppressing *S*. *frugiperda* in Kenya [18].

*S*. *frugiperda* was first detected in China on December 11th, 2018 [19], with the corn-strain of FAW invading the country [20, 21]. After moving to China from Myanmar, this invasive pest invaded 26 provinces, with Guangxi and Yunnan being the most heavily infested [20], and caused severe damage to corn, barley, soybean, peanut, tomato and tobacco in Yunnan province, China [22]. *S*. *frugiperda* larvae cause 78 and 65% damage during the seedling and flowering phases, respectively, in the peanut field [23], while 30 to 90% damage in the wheat field [24], and 10 to 80% damage in barley fields [11, 25]. This invasive pest has the potential for causing damage in tobacco crops only if the population is at a peak in the area [26].

Many research studies have been focused on the development of environment friendly control approaches for managing arthropods pests, including biopesticides [27–34], natural enemies [35, 36] and soft acaricides [37, 38] as a key components of integrated pest management approach. These environment friendly control tactics are even effective for different insect pests but work in slow manner. Hence, owing to slow action mechanism of safer control tactics, most of the farmers prefer traditional insecticide against insect pests as an emergency response to tackle invasive pests [39]. However, due to the pest’s genetic plasticity, high fecundity, and especially intense selection pressure, *S*. *frugiperda* has developed resistance to a variety of insecticide classes [40], for example, organophosphates (chlorpyriphos) [25], carbamates (carbaryl) [41], benzoylurea (lufenuron) [42], spinosyn (spinosad) [43], and diamides (chlorantraniliprole) [44].

The application of novel synthetic insecticides is an effective emergency-based control method that might be an essential component of integrated pest management strategies to tackle invasive *S*. *frugiperda* in China [45]. Because of this, evaluating the efficiency of synthetic insecticides against *S*. *frugiperda* laboratory populations is a top priority [46]. Farmers and agricultural managers have no experience dealing with *S*. *frugiperda*, which is necessary for the development of effective management strategies [47]. To meet the food demands of a growing population, the maize production systems deployed by smallholders in China have tended towards the highly excessive use of chemicals, which have caused severe environmental impacts [48]. However, there is little evidence on the efficiency of locally available synthetic insecticides against *S*. *frugiperda*.

Hence, the objective of the present work is to evaluate the laboratory efficacy of selected synthetic insecticides against new invasive pests in China to develop an emergency-based approach by novel insecticides for minimizing yield losses by suppressing this notorious pest in China and other affected countries.

## Materials and methods

### Insects

Individuals of fall armyworms were reared on an artificial diet containing, 26.0g of agar, 1.0g of choline chloride, 0.2g of myo-inositol, 2.0g of sorbic acid and propyl 4-hydroxy benzoate, 0.1g of streptomycin sulphate and penicillin GNa salt, 8.0g of ascorbic acid, 100g of soyabean, 80g of wheat bran, 26g of yeast extract, 8g of casein and 100 ml of double distilled water. Fall armyworms were kept at 25 ± 2°C and were subject to a 12:12 (light: dark) h photoperiod and relative humidity (RH) between 50 and 70% in an environmental growth chamber at the Institute of Zoology, Guangdong Academy of Sciences, Guangzhou, China.

### Synthetic insecticides

This experimental study tested the efficacy of seven synthetic insecticides from four different chemical groups to second instar *S*. *frugiperda* larvae after being purchased from different manufacturers. Chemical groups, active component percentages, formulation type, and supplier’s information are captured in Table 1. The five serial concentrations of each synthetic insecticide were generated prior to the bioassay using repeated dilutions with distilled water.

**Table 1:** Insecticide formulations tested against *S*. *frugiperda* larvae including active ingredients, formulation type, concentrations, chemical group and respective mode of action.

Active Ingredient	Formulation (g liter^-1^)	Chemical Group ^a^	Manufacturer	IRAC MOA^b^
**Spinetoram**	25% WG	5, Spinosyns	Dow Agrosciences	Nicotinic acetylcholine receptor (nAChR) allosteric modulators site I
**Spinosad**	25 SC	5, Spinosyns	Dow Agrosciences	Nicotinic acetylcholine receptor (nAChR) allosteric modulators site I
**Lambda-cyhalothrin**	10% EW	3A, Pyrethroids	Jiangsu Yangnong Chemicals	Sodium channel modulators
**Cypermethrin**	10% EC	3A, Pyrethroids	Jiangsu Yangnong Chemicals	Sodium channel modulators
**Bifenthrin**	100 EC	3A, Pyrethroids	Jiangsu Yangnong Chemicals	Sodium channel modulators
**Abamectin**	5% EC	6A, Avermectins	Beijing Kefa Weiye Chemicals	Glutamate-gated chloride (GluCl) allosteric modulators
**Broflanilide**	100 SC	28A, Diamides	DuPont, USA	Ryanodine receptor (RyR) modulators

### Bioassay

The artificial diet mix method assessed the efficiency of seven synthetic insecticides against second instar *S*. *fruigiperda* larvae. The insecticide stock solutions were prepared by dissolving each in 1 ml acetone; and then diluted with distilled water to prepare five serial concentrations (Conc.1 to 5) for each insecticide i.e., 10.0, 5.0, 2.5, 1.25, 0.625 mg liter^-1^, respectively, for spinetoram, spinosad, lambda-cyhalothrin, cypermethrin, bifenthrin, abamectin and broflinilide. The artificial diet mixed insecticides for each concentration were placed in clean rectangular plastic boxes (28 × 17 × 18 cm), with perforated lid. Second instar *S*. *frugiperda* larvae collected from culture were released onto artificial diet. Larval mortality was assessed at 24, 48, and 72 h after exposure to the treated artificial diet using a camel hairbrush. The larvae that responded to the gentle touch of a camel hair brush were considered alive, while those who failed to move were considered dead. Thirty larvae were considered one replicate; five replicates were performed for each insecticide concentration, while an artificial diet mixed with water was used as the control.

### Statistical analysis

The mean percentage larval mortality and means numbers of *S*. *frugiperda* larvae obtained from the laboratory were subjected to one-way analysis of variance (ANOVA) using a generalized linear model. The percent larval mortality data of synthetic insecticides was transformed using an arcsine transformation to normalize the variance [49]. The significant level was set at (*p* < 0.05), and the means were separated using Tukey’s honest significant different test. Lethal concentrations (LC_50_), 95% confidence limits (CLs), slope, and chi-square (χ*2*) were calculated using the POLO Plus software (version 2.0, LeOra Software, Berkeley, CA, USA), while degree of freedom (df) and *p* value were calculated using the IBM SPSS statistics software package version 23.0 (Armonk, New York, USA). The letters were calculated using software Statistix, version 8.1. Relative potency ratios to estimate the potency of the active ingredients were calculated as the LC_50_ of the least toxic compound divided by the LC_50_ of the most toxic compound [40].

## Results

### Efficacy of synthetic insecticides against second instar *S*. *frugiperda* larvae

There was a significant difference among synthetic insecticides causing larval mortality to second instar *S*. *frugiperda* at concentration 1 (F = 47.32; df = 20; *p* < 0.0000) at 24 h post-treatment. The broflanilide, abamectin and spinetoram outperformed all the other insecticides by causing larval mortality of 70.7, 68.0 and 67.3%, respectively, followed by lambda-cyhalothrin (65.3%) to second instar *S*. *frugiperda*, while, lowest larval mortality was observed with bifenthrin (58.0%) as compared to control (4.7%). However, all other insecticides caused 60.7 to 61.3% larval mortality. At concentration 2 (F = 22.82; df = 7; *p* < 0.0000), broflanilide and abamectin causing larval mortality of 59.63% followed by 57.3 and 56.0% for lambda-cyhalothirn and spinetoram, respectively. The lowest larval mortality was observed for bifenthrin (47.3%) compared to control (3.7%). However, all other insecticides caused 48.0 to 49.0% larval mortality. At concentration 3 (F = 11.33; df = 7; *p* < 0.0000), abamectin and lambda-cyhalothrin cause highest larval mortality of 48.0% followed by 46.0 and 44.7% for broflanilide and spinetoram, respectively. The lowest larval mortality was observed with spinosad (35.3%), compared to control (2.5%), while, all other insecticides caused larval mortality of 36.0%. At concentration 4 (F = 10.11; df = 7; *p* < 0.0000), abamectin and lambda-cyahlothrin caused larval mortality of 39.3and 38.0%, respectively, followed by broflanilide (37.3%). The lowest larval mortality of 23.3% was observed for bifenthrin and spinosad as compared to 1.5% in control. However, all other insecticides caused 27.3 to 32.7% larval mortality. At concentration 5 (F = 11.25; df = 7; *p* < 0.0000), broflanilide caused highest larval mortality of 32.0% followed by 27.3 and 26.0% for abamectin and cypermethrin, respectively, as compared to 0.0% in the control. The lowest larval mortality was observed with spinosad (10.7%). However, all other insecticides caused 11.3 to 21.3% larval mortality were shown in Table 2.

**Table 2:** Percent mortality of second instar *S*. *frugiperda* larvae to synthetic insecticides at 24 h post-treatment.

Percent Mortality (± SEM)					
Insecticide	10 mg L^-1^	5 mg L^-1^	2.5 mg L^-1^	1.25 mg L^-1^	0.625 mg L^-1^
**Spinetoram**	67.3 ± 1.2a A	56.0 ± 0.7a AB	44.7 ± 1.0a BC	32.7 ± 0.6a CD	21.3 ± 0.7ab D
**Spinosad**	61.3 ± 1.1a A	48.7 ± 1.0a B	35.3 ± 0.5a C	23.3 ± 0.8a D	10.7 ± 0.4bc E
**Lambda-cyhalothrin**	65.3 ± 1.5a A	57.3 ± 2.2a A	48.0 ± 0.9a AB	38.0 ± 0.9a BC	26.0 ± 0.6a C
**Cypermethrin**	60.7 ± 0.7a A	48.0 ± 1.0a B	36.0 ± 0.9a C	27.3 ± 0.4a CD	20.0 ± 1.0ab D
**Bifenthrin**	58.0 ± 0.8a A	47.3 ± 0.7a AB	36.0 ± 1.8a BC	23.3 ± 2.3a CD	11.3 ± 1.3bc D
**Abamectin**	68.0 ± 0.5a A	59.3 ± 1.2a AB	48.0 ± 0.7a BC	39.3 ± 0.8a C	27.3 ± 0.6a D
**Broflanilide**	70.7 ± 0.9a A	59.3 ± 1.1a AB	46.0 ± 2.5a BC	37.3 ± 1.1a C	32.0 ± 1.2a C
**Control**	4.7 ± 0.2b A	3.7 ± 0.1b A	2.5 ± 0.2b A	1.5 ± 0.5b A	0.0 ± 0.2c A
**ANOVA**					
***F***	47.32	22.82	11.33	10.11	11.25
***df***	7	7	7	7	7
***P<***	0.0000	0.0000	0.0000	0.0000	0.0000

Means ± SE within a column of each insecticide and row of each concentration followed by the same letters are not significantly different at *P* = 0.05 (Tukey’s test). Lower case letters immediately following values represent comparisons within a column and capital letters represent comparisons within a row. Means were calculated from five repetitions each comprising *n* = 30 larvae.

The significant difference was observed among five serial concentrations with spinetoram (F = 41.12; df = 4; *p* < 0.0000), spinosad (F = 56.07; df = 4; *p* < 0.0000), lambda-cyhalothrin (F = 11.86; df = 4; *p* < 0.0000), cypermethrin (F = 34.08; df = 4; *p* < 0.0000), bifenthrin (F = 13.60; df = 4; *p* < 0.0000), abamectin (F = 35.76; df = 4; *p* < 0.0000), and broflanilide (F = 10.50; df = 4; *p* < 0.0000) caused larval mortality to second instar *S*. *frugiperda* at 24 h post-treatment as shown in Table 2.

There was a significant difference among synthetic insecticides causing larval mortality to second instar *S*. *frugiperda* at concentration 1 (F = 66.08; df = 7; *p* < 0.0000) 48 h post-treatment. Spinetoram and broflanilide outperformed all the other insecticides by causing larval mortality of 87.3 and 84.7%, respectively, followed by abamectin, spinosad and lambda-cyhalothrin causing 80.7, 80.0 and 79.3% mortality to second instar *S*. *frugiperda*, as compared to control (5.30%). However, all other insecticides caused 72.0 to 75.3% larval mortality. At concentration 2 (F = 39.52; df = 7; *p* < 0.0000), spinetoram causing larval mortality of 74.0% followed by 73.3 and 72.0% for broflanilide and abamectin, respectively as compared to control (3.7%). However, cypermethrin and bifenthrin caused larval mortality of 63.3% while, 66.7% larval mortality for spinosad. At concentration 3 (F = 20.68; df = 7; *p* < 0.0000), broflanilide caused highest larval mortality of 65.0% followed by 60.0 and 60.7% for abamectin and spinetoram, respectively, compared to control (2.5%). Spinosad, lambda-cyahlothrin and bifenthrin caused 54.7% larval mortality while 52.0% larval morality for cypermethrin. At concentration 4 (F = 15.15; df = 7; *p* < 0.0000), broflanilide and abamectin caused larval mortality of 55.3 and 50.0%, respectively, followed by 47.3 and 46.7% for spinetoram and lambda-cyhalothrin as compared to 1.5% in control. However, all other insecticides caused 39.3 to 42.7% larval mortality. At concentration 5 (F = 15.33; df = 7; *p* < 0.0000), broflanilide caused highest larval mortality of 47.3% followed by 40.0% for abamectin as compared to 0.0% in the control. The lowest larval mortality was observed with spinosad (24.7%). However, all other insecticides caused 30.0 to 34.7% larval mortality 78 h post-treatment as shown in Table 3.

**Table 3:** Percent cumulative mortality of second instar *S*. *frugiperda* larvae to synthetic insecticides at 48 h post-treatment.

Percent Mortality (± SEM)					
Insecticide	10 mg L^-1^	5 mg L^-1^	2.5 mg L^-1^	1.25 mg L^-1^	0.625 mg L^-1^
**Spinetoram**	87.3 ± 0.6a A	74.0 ± 1.2a B	60.7 ± 0.9a C	47.3 ± 0.4a D	34.0 ± 1.1abc E
**Spinosad**	80.0 ± 1.4ab A	66.7 ± 0.8a B	54.7 ± 0.7a B	39.3 ± 0.6a C	24.7 ± 0.5c D
**Lambda-cyhalothrin**	79.3 ± 1.3ab A	67.3 ± 1.5a AB	54.7 ± 1.0a BC	46.7 ± 0.7a CD	34.7 ± 0.9abc D
**Cypermethrin**	75.3 ± 0.9ab A	63.3 ± 1.6a AB	52.0 ± 1.2a BC	42.7 ± 0.7a CD	33.3 ± 0.7abc D
**Bifenthrin**	72.0 ± 1.1b A	63.3 ± 0.6a AB	54.7 ± 2.2a ABC	42.0 ± 2.8a BC	30.0 ± 1.0bc C
**Abamectin**	80.7 ± 0.7ab A	72.0 ± 1.5a AB	60.0 ± 0.7a BC	50.0 ± 0.6a CD	40.0 ± 1.1ab D
**Broflanilide**	84.7 ± 1.1ab A	73.3 ± 0.5a AB	65.3 ± 1.8a BC	55.3 ± 1.4a BC	47.3 ± 1.4a C
**Control**	5.30 ± 0.2c	4.10 ± 0.1b	3.00 ± 0.1b	1.00 ± 0.2b	0.0 ± 0.0d
**Anova**					
***F***	66.08	39.52	20.68	15.15	15.53
***df***	7	7	7	7	7
***P<***	0.0000	0.0000	0.0000	0.0000	0.0000

Means ± SE within a column of each insecticide and row of each concentration followed by the same letters are not significantly different at *P* = 0.05 (Tukey’s test). Lower case letters immediately following values represent comparisons within a column and capital letters represent comparisons within a row. Means were calculated from five repetitions each comprising *n* = 30 larvae.

The significant difference was observed among five serial concentrations with spinetoram (F = 53.61; df = 4; *p* < 0.0000), spinosad (F = 56.83; df = 4; *p* < 0.0000), lambda-cyhalothrin (F = 21.58; df = 4; *p* < 0.0000), cypermethrin (F = 21.02; df = 4; *p* < 0.0000), bifenthrin (F = 8.00; df = 4; *p* < 0.0000), abamectin (F = 24.43; df = 4; *p* < 0.0000), and broflanilide (F = 11.13; df = 4; *p* < 0.0000) caused larval mortality to second instar *S*. *frugiperda* at 48 h post-treatment as captured in Table 3.

There was a significant difference among synthetic insecticides causing larval mortality to second instar *S*. *frugiperda* at concentration 1 (F = 121.99; df = 7; *p* < 0.0000) 72 h post-treatment. Spinetoram and broflanilide outperformed all the other insecticides by causing larval mortality of 93.3 and 91.3%, respectively, followed by spinosad and abamectin causing 89.3 and 87.3% larval mortality to second instar *S*. *frugiperda*, as compared to control (5.30%). However, all other insecticides caused (>80.0%) larval mortality. At concentration 2 (F = 35.79; df = 7; *p* < 0.0000), broflanilide and spinetoram causing larval mortality of 82.0 and 81.3%, respectively, followed by abamectin (78.7%), as compared to control (3.7%). However, all other insecticides caused (>70.0%) larval mortality. At concentration 3 (F = 23.34; df = 7; *p* < 0.0000), broflanilide caused highest larval mortality of 71.3% followed by 67.3 and 66.7% for spinetoram and abamectin, respectively, compared to control (2.5%). However, all other insecticides caused (>60.0%) larval mortality. At concentration 4 (F = 19.77; df = 7; *p* < 0.0000), broflanilide caused highest larval mortality of 60.7%, followed by 57.3 and 56.7% for cypermethrin and abamectin as compared to 2.5% in control. However, all other insecticides caused (>50.0%) larval mortality. At concentration 5 (F = 15.33; df = 7; *p* < 0.0000), broflanilide caused highest larval mortality of 53.3% followed by 47.3 and 46.7% for abamectin and cypermethrin as compared to 0.0% in the control. The lowest larval mortality was observed with spinosad (32.7%). However, all other insecticides caused (>40.0%) larval mortality to second instar *S*. *frugiperda* at 72 h post-treatment in Table 4.

**Table 4:** Percent cumulative mortality of second instar *S*. *frugiperda* larvae to synthetic insecticides at 72 h post-treatment.

Percent Mortality (± SEM)					
Insecticide	10 mg L^-1^	5 mg L^-1^	2.5 mg L^-1^	1.25 mg L^-1^	0.625 mg L^-1^
**Spinetoram**	93.3 ± 0.5a A	81.3 ± 1.0a B	67.3 ± 0.7a C	55.3 ± 0.4a D	42.0 ± 1.2ab E
**Spinosad**	89.3 ± 0.7ab A	73.3 ± 1.5a B	60.7 ± 1.2a BC	47.3 ± 0.4a C	32.7 ± 0.5b D
**Lambda-cyhalothrin**	86.0 ± 0.9ab A	74.0 ± 1.6a AB	63.3 ± 1.1a BC	54.7 ± 0.8a CD	42.7 ± 1.1ab D
**Cypermethrin**	82.0 ± 0.7ab A	71.3 ± 2.1a AB	65.3 ± 0.2a B	57.3 ± 0.9a BC	46.7 ± 0.9ab C
**Bifenthrin**	80.0 ± 1.0b A	70.0 ± 0.4a AB	62.7 ± 2.6a AB	54.7 ± 2.7a B	45.3 ± 0.7ab B
**Abamectin**	87.3 ± 0.7ab A	78.7 ± 1.6a AB	66.7 ± 0.7a BC	56.7 ± 0.9a CD	47.3 ± 1.1a D
**Broflanilide**	91.3 ± 1.1ab A	82.0 ± 0.9a AB	71.3 ± 1.9a BC	60.7 ± 1.5a C	53.3 ± 1.4a C
**Control**	6.7 ± 0.0c A	4.5 ± 0.0b A	3.5 ± 0.0b A	2.5 ± 0.0b A	0.0 ± 0.0c A
**ANOVA**					
***F***	121.99	35.79	23.34	19.77	20.66
***df***	7	7	7	7	7
***P<***	0.0000	0.0000	0.0000	0.0000	0.0000

Means ± SE within a column of each insecticide and row of each concentration followed by the same letters are not significantly different at *P* = 0.05 (Tukey’s test). Lower case letters immediately following values represent comparisons within a column and capital letters represent comparisons within a row. Means were calculated from five repetitions each comprising *n* = 30 larvae.

The significant difference was observed among five serial concentrations with spinetoram (F = 56.40; df = 4; *p* < 0.0000), spinosad (F = 47.29; df = 4; *p* < 0.0000), lambda-cyhalothrin (F = 20.17; df = 4; *p* < 0.0000), cypermethrin (F = 12.48; df = 4; *p* < 0.0000), bifenthrin (F = 5.17; df = 4; *p* < 0.0000), abamectin (F = 20.94; df = 4; *p* < 0.0000), and broflanilide (F = 11.06; df = 4; *p* < 0.0000) caused larval mortality to second instar *S*. *frugiperda* at 72 h post-treatment in Table 4.

### Susceptibility of the second instar *S*. *frugiperda* larvae to synthetic insecticides

The toxicity regression equations, lethal concentration 50 (LC_50_), and toxicity index were estimated based on larva mortality at 24 h post-treatment in Table 5. The results presented the toxicity of seven synthetic insecticides, including broflanilide, abamectin and spinetoram were found to be lower than those of the other four insecticides including spinosad, lambda-cyhalothrin, cypermethrin and bifenthrin. The broflanilide and abamectin were showed lowest LC_50_ values of 0.825 and 1.223 mg liter^-1^, respectively, followed by spinetoram (1.408 mg liter^-1^), while, bifenthrin and spinosad were showed highest LC_50_ value of 2.123 and 2.122 mg liter^-1^, respectively. The obtained results demonstrated that broflanilide and abamectin outperformed among all other insecticides causing toxicity to the second instar *S*. *frugiperda* larvae. Based on the toxicity index at the LC_50_ level, broflanilide was the most effective insecticide (100%), followed abamectin (67.46%), while, the least toxicity index of 38.86 and 38.88% was observed in bifenthrin and spinosad, respectively. The toxicity index of 41.54 to 58.59% for all other insecticides to second instar *S*. *frugiperda* larvae at 24 h post-treatment in Table 5.

**Table 5:** Susceptibility of the second instar *S*. *frugiperda* larvae to synthetic insecticides at 24 h post-treatment.

Fit of probe line							
Insecticides	Slope ± SE	chi-sq. (χ*2*)	df	*P* value	LC_50_ (95% CL) (mg L^-1^)	Reg. equation (*y* = *a* + *bx*)	Toxicity Index
**Spinetoram**	1.25 ± 0.12	0.77	3	0.89	1.41 (1.13–1.69) ^defg^	−0.19+1.27x	58.59
**Spinosad**	1.24 ± 0.12	0.23	3	0.97	2.12 (1.76–2.53) ^cdef^	−0.41+1.24 x	38.88
**Lambda-cyhalothrin**	0.97 ± 0.11	0.73	3	0.87	1.63 (1.25–2.04) ^bcde^	−0.21+0.98 x	50.49
**Cypermethrin**	0.91 ± 0.11	0.46	3	0.93	1.99 (1.53–2.51) ^bcd^	−0.27+0.91 x	41.54
**Bifenthrin**	0.91 ± 0.11	0.47	3	0.93	2.12 (1.65–2.68) ^c^	−0.30+0.91 x	38.86
**Abamectin**	0.93 ± 0.11	0.20	3	0.98	1.22 (0.88–1.57) ^ab^	−0.08+0.93 x	67.46
**Broflanilide**	0.87 ± 0.12	0.95	3	0.81	0.82 (0.52–1.12) ^a^	0.07+0.88 x	100

SE, df and CL indicate standard error, degrees of freedom and confidence limits, respectively. The LC_50_ values of each tested insecticide followed by different lower-case letters in the same column indicate significantly different when non-overlapping with each other in the corresponding 95% confidence limits (P < 0.05). Toxicity Index ₌ LC_50_ of the most effective compound/LC_50_ of the other tested compound × 100.

The results presented the toxicity of seven synthetic insecticides, including broflanilide and abamectin were found to be lower than those of the other five synthetic insecticides, spinetoram, spinosad, lambda-cyhalothrin, cypermethrin and bifenthrin. The broflanilide and abamectin were showed lowest LC_50_ values of 2.664 and 2.796 mg liter^-1^, respectively, followed by lambda-cyhalothrin (3.123 mg liter^-1^), while, bifenthrin and cypermethrin were showed highest LC_50_ value of 5.942 and 5.505 mg liter^-1^, respectively. The obtained results demonstrated that broflanilide and abamectin outperformed among all other insecticides causing toxicity to the second instar *S*. *frugiperda* larvae. Based on the toxicity index at the LC_50_ level, broflanilide and abamectin were the most effective insecticide by indicating the toxicity index of 100 and 95.28% followed by lambda-cyhalothrin and spinetoram were showed toxicity index of 85.30 and 75.30%, respectively, while, the least toxicity index of 44.83% was observed in bifenthrin. The toxicity index of (> 48.0%) for all other insecticides to second instar *S*. *frugiperda* larvae at 42 h post-treatment in Table 6.

**Table 6:** Susceptibility of the second instar *S*. *frugiperda* larvae to synthetic insecticides at 48 h post-treatment.

Fit of probe line							
Insecticides	Slope ± SE	chi-sq. (χ*2*)	df	*P* value	LC_50_ (95% CL) (mg L^-1^)	Reg. equation (*y* = *a* + *bx*)	Toxicity Index
**Spinetoram**	1.02 ± 0.11	0.10	3	0.99	3.54 (2.86–4.50) ^abd^	−0.56+1.02x	75.30
**Spinosad**	1.22 ± 0.12	0.91	3	0.82	5.43 (4.44–6.96) ^cdf^	−0.91+1.25 x	49.08
**Lambda-cyhalothrin**	0.85 ± 0.11	0.49	3	0.92	3.12 (2.43–4.13) ^e^	−0.42+0.85 x	85.30
**Cypermethrin**	0.92 ± 0.11	0.35	3	0.95	5.50 (4.25–7.78) ^cd^	−0.68+0.92 x	48.39
**Bifenthrin**	1.13 ± 0.12	1.35	3	0.72	5.94 (4.76–7.91) ^c^	−0.89+1.15 x	44.83
**Abamectin**	0.88 ± 0.11	0.24	3	0.97	2.80 (2.19–3.62) ^ab^	−0.39+0.88 x	95.28
**Broflanilide**	0.86 ± 0.11	1.44	3	0.70	2.66 (2.07–3.46) ^a^	−0.36+0.86 x	100

SE, df and CL indicate standard error, degrees of freedom and confidence limits, respectively. The LC_50_ values of each tested insecticide followed by different lower-case letters in the same column indicate significantly different when non-overlapping with each other in the corresponding 95% confidence limits (P < 0.05). Toxicity Index ₌ LC_50_ of the most effective compound/LC_50_ of the other tested compound × 100

The results presented the toxicity of seven synthetic insecticides, including broflanilide and cypermethrin were found to be lower than those of the other five synthetic insecticides, spinetoram, spinosad, lambda-cyhalothrin, abamectin and bifenthrin. Broflanilide and cypermethrin were showed lowest LC_50_ values of 0.606 and 0.783 mg liter^-1^, respectively, followed by abamectin (0.803 mg liter^-1^), while, spinosad and lambda-cyhalothrin were showed highest LC_50_ value of 1.439 and 1.001 mg liter^-1^, respectively. The obtained results demonstrated that broflanilide, cypermethrin and abamectin outperformed among all other insecticides causing toxicity to the second instar *S*. *frugiperda* larvae. Based on the toxicity index at the LC_50_ level, broflanilide and cypermethrin were the most effective insecticide by indicating the toxicity index of 100 and 77.39%, respectively, followed by abamectin (75.47%), while, the least toxicity index of 42.11% was observed in spinosad. The toxicity index of (> 60.0%) for all other insecticides to second instar *S*. *frugiperda* larvae at 72 h post-treatment in Table 7.

**Table 7:** Susceptibility of the second instar *S*. *frugiperda* larvae to synthetic insecticides at 72 h post-treatment.

Fit of probe line							
Insecticides	Slope ± SE	chi-sq. (χ*2*)	df	*P* value	LC_50_ (95% CL) (mg L^-1^)	Reg. equation (*y* = *a* + *bx*)	Toxicity Index
**Spinetoram**	1.33 ± 0.13	2.30	3	0.51	0.98 (0.76–1.19) ^cdefg^	6.68+1.38 x	62.09
**Spinosad**	1.32 ± 0.12	1.93	3	0.59	1.44 (1.18–1.71) ^f^	−0.21+1.35 x	42.11
**Lambda-cyhalothrin**	0.10 ± 0.12	1.03	3	0.79	1.00 (0.71–1.29) ^cde^	−3.01+1.01 x	60.54
**Cypermethrin**	0.78 ± 0.11	0.62	3	0.89	0.80 (0.54–1.06) ^bcd^	−0.09+1.01 x	75.47
**Bifenthrin**	0.77 ± 0.11	0.34	3	0.95	0.91 (0.55–1.26) ^abc^	0.03+0.77 x	66.89
**Abamectin**	0.10 ± 0.12	0.73	3	0.87	0.80 (0.54–1.06) ^ab^	0.09+1.01 x	78.29
**Broflanilide**	1.03 ± 0.12	1.79	3	0.62	0.61 (0.38–0.82) ^a^	0.22+1.06 x	100

SE, df and CL indicate standard error, degrees of freedom and confidence limits, respectively. The LC_50_ values of each tested insecticide followed by different lower-case letters in the same column indicate significantly different when non-overlapping with each other in the corresponding 95% confidence limits (P < 0.05). Toxicity Index ₌ LC_50_ of the most effective compound/LC_50_ of the other tested compound × 100

## Discussion

Pesticide residue levels in produce have become stricter due to a growing demand for high-quality and safe food. Synthetic insecticides although have residual effects but could be used after evaluating the optimum dose level with least residual effects as an emergency control of many arthropods especially lepidopteran pests.

The present study was conducted to assess the susceptibility of second instar larvae of Fall armyworm *Spodoptera frugiperda* (Lepidoptera: Noctuidae) at five different concentrations after different time exposures to seven synthetic insecticides belong to different chemical groups including two spinosyns, three pyrethroids, one avermectin and one diamides in China. The present study results revealed that all of the tested synthetic insecticides showed significant efficacy against second instar *S*. *frugiperda* larvae. Recently, many researchers around the world have been investigating the different management strategies against FAW in field and laboratory to develop registered pesticides as an emergency approach including India, Africa, Brazil and Indonesia [50–53], where this invasive pest caused severe damage to maize crop. However, this is the first study to our knowledge reporting the laboratory toxicity of seven synthetic insecticides to *S*. *frugiperda* larvae.

The results of present study revealed that second instar *S*. *frugiperda* larvae was susceptible to seven synthetic insecticides; however, spinetoram, broflanilide and spinosad were caused highest larval mortality 72 h post-treatment. The present study results align with Kulye et al. [51], who observed *S*. *frugiperda* susceptible to spinetoram across field samples. The highest toxicity of spinetoram in causing larval mortality to second instar *S*. *frugiperda* larvae [50]. Spinosad and abamectin displayed lower resistance and still effectively manage fall armyworm resistance in Puerto Rico [40]. Another study reported that cypermethrin caused 76.7% mortality to the larvae of corn earworm, *Helicoverpa zea* (Boddie) (Lepidoptera: Noctuidae) [53]. According to a recent study, the beet armyworm, *Spodoptera exigua* (Hübner) (Lepidoptera: Noctuidae), was highly susceptible to broflanilide. Hence, broflanilide could be an important new tool alternative to synthetic insecticides to control lepidopteran pests [54].

Another study demonstrated that broflanilide is expected to become a prominent insecticide because it is effective against *S*. *litura* with resistance to cyclodienes and fipronil [55]. The resistance was higher in bifenthrin to American bollworm, *Helicoverpa armigera* (Hübner) (Lepidoptera: Noctuidae) [56]. The results of present study are in lined with [41] reported that more than 80% mortality to third instar *S*. *frugiperda* larvae at 96 h after application of spinosad. Another study demonstrated that spinosad showed highest toxicity against *S*. *frugiperda* larvae [57]. The higher concentrations and time exposure of synthetic insecticides increase larval mortality of *S*. *frugiperda* both in the field as well as in laboratory assay [57, 58].

*S*. *frugiperda* was resistant to lambda-cyhalothrin due to continuous application of conventional insecticides in Colombia and China [59, 60]. Some of the pests such as the diamondback moth, *Plutella xylostella* (Lepidoptera: Plutellidae) [61] and two-spotted spider mites, *Tetranychus urticae* (Trombidiformes: Tetranychidae) [62], have become resistant to abamectin while spinetoram still proved to be efficient in suppressing FAW in field trials. Even though chemical pesticides are critical in FAW control, the insect has become resistant to many of them as a result of their widespread usage.

The results of present study are in lined with [50] reported that the LC_50_ values of spinetoram were lower than lambda-cyhalothrin. The findings of the present study in lined with Hardke et al. [63] who reported that LC_50_ value of spinetoram was significantly lower as compared to the indoxacarb. Although synthetic insecticides are effective against *S*. *frugiperda*, but also have harmful effect on human and ecosystem due to a lack of appropriate information about insecticides dose recommendation and safety precautions which is an important issue for the application of synthetic insecticide in the field [64]. This situation suggests that there is a dire need to develop novel insecticides with optimum dose as a component of IPM.

The recent invasion of an invasive pest has alarmed many affected countries such as Africa, Ethiopia, India [18]. Therefore, there is an urgent need of insecticides spraying program as an emergency response in *S*. *frugiperda* invaded countries, especially in the maize fields, to protect crop damage and prevent the further expansion of the invasive pest. Farmers are applying unregistered synthetic insecticides in many countries, including Ethiopian and Kenya [65]. This work adds to our understanding of the efficacy of novel insecticides in the management of *S*. *frugiperda*. These pesticides recommend only as a last option in treating lepidopteran pests as part of an integrated pest management strategy.

## Conclusions

Since very low resistance levels have been determined with broflanilide, cypermethrin and abamectin, hence, these chemicals should therefore be used cautiously and appropriately in management plans to retain their effectiveness for as long as possible. The highest discriminating concentrations of novel synthetic insecticides dramatically increase the cumulative larval mortality of early instar *S*. *frugiperda*. The findings of present study suggested that larval mortality of early instar significantly increase with increasing concentrations. Additionally, these insecticides could be used on an emergency basis at recommended dose against *S*. *frugiperda* larvae after further investigation on their efficacy are obtained in the field. The results of this study provided valuable information for choosing alternative insecticides substitute of *S*. *frugiperda* resistant insecticides.
